# Low Utilization of Dietetic Support and Determinants of Lifestyle Modification Among Women With Polycystic Ovarian Syndrome: A Cross-Sectional Study

**DOI:** 10.7759/cureus.108939

**Published:** 2026-05-15

**Authors:** Sivaranjani P, Aprajita A

**Affiliations:** 1 Obstetrics and Gynaecology, Jawaharlal Institute of Postgraduate Medical Education and Research (JIPMER), Puducherry, IND; 2 Medicine, All India Institute of Medical Sciences, Kalyani, Kalyani, IND

**Keywords:** allied health professional, dietic support, lifestyle modification, polycystic ovary syndrome (pcos), weight reduction

## Abstract

Introduction

Polycystic ovarian syndrome (PCOS) is one of the most common endocrine disorders among women of reproductive age. It is associated with metabolic and reproductive complications, and lifestyle modification plays an important role in its management. However, adherence to these measures remains suboptimal.

Objective

The objective of this study was to assess the facilitators and barriers to lifestyle modification among women with PCOS.

Methods

This cross-sectional study was conducted in the gynecology outpatient department of a tertiary care institute over three months. Women aged 18-40 years diagnosed with PCOS based on Rotterdam criteria were included. Data were collected using a structured questionnaire assessing sociodemographic characteristics, symptomatology, dietary habits, physical activity, and factors influencing lifestyle modification. Statistical analysis was performed using SPSS software, version 22.0 (IBM Corp., Armonk, NY).

Results

A total of 169 women were included. The median age was 28 years, and the median Body Mass Index (BMI) was 26 kg/m². Overall, 72.2% of participants did not achieve weight reduction, while 27.8% achieved ≥5 kg weight loss. Depressive mood was significantly associated with failure to reduce weight (p = 0.002), whereas concern about body image facilitated weight reduction (p = 0.032). Common barriers included fatigue (74.6%), lack of time (42.6%), and easy accessibility to junk food (56.2%). Only 3.55% of participants had consulted a dietitian.

Conclusion

Adherence to lifestyle modification among women with PCOS remains low, with multiple physical and psychological barriers identified. Utilization of dietetic support was minimal. Addressing these barriers through structured counseling and multidisciplinary care may help improve adherence and clinical outcomes.

## Introduction

Polycystic ovarian syndrome (PCOS) is the most common endocrine disorder among women of reproductive age. The prevalence of PCOS worldwide is 2.2 to 26 % [1]. The prevalence of PCOS in India is 8.2 to 22.5 % [2]. Insulin resistance is the basic pathogenesis of PCOS. PCOS women are at risk of metabolic, reproductive, and psychological disorders. And it is also associated with long-term complications like diabetes and cardiovascular disorders. The prevalence of overweight and obesity is higher in women with PCOS compared to women without PCOS. The propensity to gain weight is also higher in women with PCOS compared to women without PCOS. Weight gain and obesity further worsen the insulin resistance and the overall features of PCOS. Thus, Lifestyle management is recommended as the first line of management [3]. There exist many barriers to lifestyle modification. Women in the reproductive age group with or without PCOS face many challenges in modifying their lifestyle. In addition to the barriers associated with women without PCOS, PCOS women has increased risk of fatigue and sleep, eating disorder, depressive mood and body image distress which imposed further challenges among PCOS women to achieve Lifestyle modification [4].

Studies conducted in European countries stated family responsibility, lack of time, fatigue, poor family support, and lack of exercise facilities as barriers to lifestyle modification [5]. A recent study in 2022 involving the Indian population stated lack of knowledge, lack of time, lack of motivation, cultural and social taboos as barriers to lifestyle modification in PCOS women. One third of the study population were adherent to treatment management [6]. There exists a sparsity of literature evaluating facilitators to lifestyle modification in PCOS women in the Indian population. In this study, we aim to assess the facilitators in addition to barriers to lifestyle modification in PCOS women, which will guide us to promote lifestyle modification in our Indian population, which will improve the reproductive health and may prevent metabolic disorders like diabetes and cardiovascular disorders in the future. The objectives of this study were to assess the barriers and facilitators to lifestyle modification among women with PCOS and to evaluate the utilization of dietetic support in this population.

## Materials and methods

This cross-sectional study was conducted in the outpatient department of Gynecology at All India Institute of Medical Sciences, Kalyani, over a period of three months. Informed consent was obtained from all participants. All procedures involving human participants were performed in accordance with the ethical standards of the Institutional Ethics Committee (IEC/AIIMS/Kalyani/Meeting/2023/017) and with the Declaration of Helsinki and its later amendments. Consecutive consenting women were recruited into the study. Women aged 18-40 years diagnosed with PCOS based on the Rotterdam criteria for PCOS were included. Women diagnosed within the last three months and those aged less than 18 years were excluded.

Data were collected using a structured questionnaire developed by the investigators following a review of the relevant literature. The questionnaire was reviewed by subject experts in the Department of Obstetrics and Gynecology for content validity. It was subsequently pretested on a small group of women with PCOS (n = 15) to assess clarity, feasibility, and relevance. Minor modifications were made in response to feedback. However, formal reliability testing was not performed, which is a limitation of the study.

Sample size was calculated using OpenEpi software (www.OpenEpi.com). Assuming a prevalence of PCOS of 15% (based on previously reported estimates ranging from 8.2% to 22.5%) [7], with a 95% confidence level and absolute precision of 5%, the minimum sample size was calculated as 161. After accounting for a 10% non-response rate, the final sample size was estimated to be 177. As this was an exploratory study, the sample size based on the estimated prevalence of PCOS was used as an approximate guide rather than derived from a single primary outcome measure. However, 169 participants were included in the final analysis due to incomplete responses and non-consent among a small proportion of eligible participants during the study period.

Data were entered into Microsoft Excel and analyzed using Jamovi (2024) software, version 2.6 (https://www.jamovi.org). Age was summarized as median, and body mass index (BMI) was categorized according to standard BMI classifications. Categorical variables were expressed as frequencies and percentages. Participants were divided into two groups based on weight reduction: women who reduced ≥5 kg (Group F) and those who did not (Group B). Achievement of at least 5 kg weight loss was selected as a pragmatic and clinically meaningful indicator of successful lifestyle modification, based on self-reported weight change and existing recommendations that even modest weight reduction may improve menstrual and metabolic outcomes in women with PCOS. Comparisons between the two groups were performed using the Chi-square test or Fisher’s exact test, as appropriate, for categorical variables. Factors related to facilitators and barriers to lifestyle modification were also analyzed using similar bivariate methods. A p-value of <0.05 was considered statistically significant.

## Results

Baseline characteristics of study participants

Age of the participants ranged from 19 to 36, with the median of 28 (IQR: 23 - 32). The majority of women belonged to an urban area (156 women, 92.3%). Most of the women studied up to the 12th standard (110 women, 65%). The majority of women were unemployed (108 women, 63.9%). Most of the women were in extended family (105 women, 62.1%). According to the Modified Kuppuswamy Scale, the majority of the women belonged to the lower middle class (59 women, 34.9%). The BMI of study participants ranged from 15.5 to 38.2, with a median of 26. 44 (IQR: 23-28) (Table 1).

**Table 1:** Baseline characteristics of study participants (N = 169)

Variable	Category	Frequency (n)	Percentage (%)
Residence	Rural	13	7.7
	Urban	156	92.3
Education	Uneducated	1	0.6
	Up to the 5th standard	2	1.2
	6th–12th standard	110	65
	Graduate	56	33.2
Occupation	Student	27	16
	Employed	34	20.1
	Unemployed	108	63.9
Family type	Extended	105	62.1
	Nuclear	64	37.9
Socioeconomic status	Lower	9	5.3
	Upper lower	57	33.7
	Lower middle	59	34.9
	Upper middle	37	21.9
	Upper	7	4.1
Body mass index (BMI)	Underweight	8	4.7
	Normal	60	35.5
	Overweight	75	44.4
	Class I obesity	23	13.6
	Class II obesity	3	1.8

Weight reduction outcomes

Among the 169 women under study, 72.18% did not reduce weight (Group B), whereas 27.8 % reduced weight (≥ 5 kg) (Group F).

Comparison of baseline characteristics between groups

A bivariate analysis compared baseline parameters between Group B and Group F. There were no significant statistical differences in demographic features between the two groups (Table 2). 

**Table 2:** Comparison of baseline characteristics between groups

Variable	Category	Achieved Weight Reduction (n=47)	Did Not Achieve Weight Reduction (n=122)	p-value	χ²
Residence	Rural	3 (6.4%)	10 (8.2%)	0.692	0.157
	Urban	44 (93.6%)	112 (91.8%)		
Education	Uneducated	0 (0%)	1 (0.8%)	0.095	12.2
	Up to 5th	2 (4.3%)	0 (0%)		
	6th–12th	30 (63.9%)	81 (67.2%)		
	Graduate	15 (31.9%)	40 (32.8%)		
Occupation	Student	6 (12.8%)	21 (17.2%)	0.498	2.38
	Employed	7 (14.9%)	27 (22.1%)		
	Unemployed	34 (72.3%)	74 (60.7%)		
Family type	Extended	31 (66.0%)	74 (60.7%)	0.524	0.405
	Nuclear	16 (34.0%)	48 (39.3%)		
Socioeconomic status	Lower	4 (8.5%)	5 (4.1%)	0.666	2.38
	Upper lower	15 (31.9%)	42 (34.4%)		
	Lower middle	18 (38.3%)	41 (33.6%)		
	Upper middle	9 (19.1%)	28 (23.0%)		
	Upper	1 (2.1%)	6 (4.9%)		

Facilitators and barriers to lifestyle modification

Factors related to facilitators and barriers to lifestyle modification were also analyzed using similar bivariate methods (Table 3).

**Table 3:** Facilitators and barriers to lifestyle modification

Factor category	Variable	Achieved Weight Reduction (n=47)	Did Not Achieve Weight Reduction (n=122)	P- value	χ²
patient-related	Easy access to junk food	25	70	0.623	0.241
	Lack of time	15	57	0.081	3.04
	Financial constraints	22	53	0.693	0.156
	Lack of motivation	23	64	0.681	0.169
	Fatigue/sleeplessness	34	92	0.681	0.169
	Depressive mood	34	55	0.002	9.82
	Body image concern	35	69	0.032	4.6
Provider-related	No lifestyle counseling	14	38	0.864	0.029
	Advised 5% weight loss regularizes menstruation	24	52	0.323	0.977
	Advised fertility benefit	27	52	0.084	3
	Dietitian consultation	0	6	0.122	2.4
Family/Peer	Family support	40	108	0.546	0.364
	Peer support	25	48	0.103	2.65

Ninety-five women (56.21%) had easy accessibility to junk food. Seventy-two women didn’t have time to exercise, which prevented 57 women from reducing their weight. The most common barrier stated by 92 women who were not able to reduce weight was “feeling fatigued and sleepy to exercise”. Thirty-five women stated that concern about their body image prompted them to lose weight. Fifty-five women stated that depressive mood was a barrier to weight loss. There was a statistical difference between the two groups in the mean depressive mood (P value =0.002). Fifty-two women were not informed by the doctor regarding lifestyle modification. Twenty-four women reported that the doctor insisted that even a 5% reduction in weight would regularize the menstrual cycle, which facilitated their weight loss. Twenty-seven women stated that the doctor advised that lifestyle modification may improve their fertility chances, which facilitated weight reduction in them. Unfortunately, only 3.55% of the total population under study had a consultation with a dietitian. Forty women stated that they had good family support, which facilitated weight reduction for them. Twenty-five women had friends undergoing lifestyle modification, which encouraged them to do the same.

Multivariable logistic regression analysis

Multicollinearity among the independent variables included in the multivariable logistic regression model was assessed using the Variance Inflation Factor (VIF) and tolerance values. All variables had low VIF values ranging from 1.02 to 1.09, with corresponding tolerance values between 0.921 and 0.979. Since all VIF values were well below the commonly accepted threshold of 5 (and tolerance values >0.1), there was no evidence of significant multicollinearity among the predictor variables included in the model. On multivariable logistic regression analysis, absence of depressive mood was independently associated with higher odds of weight reduction (AOR: 2.49; 95% CI: 1.12-5.54; p = 0.025). Among BMI categories, women with Class I obesity had significantly higher odds of weight reduction compared to overweight women (AOR: 4.25; 95% CI: 1.24-14.55; p = 0.021). Other factors, including lack of time (AOR: 1.86; p = 0.122), body image concern (AOR: 2.00; p = 0.113), and doctor advice regarding fertility benefits (AOR: 0.73; p = 0.425), were not statistically significant after adjustment (Table 4).

**Table 4:** Multivariable logistic regression analysis of predictors of weight reduction among women with PCOS Note. Estimates represent the log odds of "M = barriers" vs. "M = facilitators". SE: standard error, Z: z-score, P: p-values.

Predictor	Estimate	SE	Z	p	Odds ratio	95% Confidence Interval	
						Lower	Upper
Intercept	1.398	0.477	2.928	0.003	4.046	1.587	10.315
Lack of time:							
Yes – No	0.623	0.403	1.544	0.122	1.864	0.846	4.109
Depressive mood:							
Yes – No	-0.912	0.408	-2.235	0.025	0.402	0.181	0.894
Doctors advised that lifestyle modification improves fertility chances.							
Yes – No	-0.308	0.386	-0.797	0.425	0.735	0.345	1.567
Obesity class:							
Underweight – Overweight	0.961	1.133	0.848	0.397	2.614	0.284	24.11
Normal – Overweight	0.683	0.431	1.583	0.113	1.979	0.85	4.608
Class I Obesity – Overweight	1.446	0.628	2.302	0.021	4.247	1.24	14.549
Class II Obesity – Overweight	0.806	1.289	0.625	0.532	2.238	0.179	27.966
I am worried about my body image.							
Yes – No	-0.693	0.437	-1.585	0.113	0.5	0.212	1.178

## Discussion

Of the total, 92.3% of study participants belonged to an urban population. This high reporting of PCOS among the urban population is supported by another study conducted in India. An increased number of urban women may be due to the easy availability and accessibility of junk foods and a sedentary lifestyle. On the contrary, rural women are comparatively physically active due to the lack of a proper transport system and machinery. In a study conducted among the Indian population, 34.9% of participants belonged to the lower middle class, whereas the majority of PCOS women belonged to the upper middle class. Of the total, 44.7 % were overweight, and 35.3 % had a normal BMI, in contrast to other studies in which almost three-fourths of the population had a normal BMI [8].

In our study, 58.68% were nonadherent to the dietary advice given by the gynecologist, which is similar to other studies conducted among an Indian population [9]. This may be due to the easy availability of junk foods, which 56.21 % of the present study participants had. Only 3.55% of the participants had a consultation with a dietitian. Lifestyle modification being the most integral part of PCOS management, it was surprising to observe that only 3.55% of the participants had a consultation with a dietitian. Pritchard et al. found that collaboration of a general practitioner and a dietitian led to successful lifestyle modification when compared to general practitioner attempts alone. Other studies also found that interaction between the general practitioner and the dietitian led to better results in lifestyle modification [10,11]. But there is also limited dietitian consultation in other studies, as in our study [12,13]. There is a need to emphasize referrals to dietitians in PCOS women, a necessity to increase the provision of dietitians in health care centers, including urban, rural, and remote areas, ensuring health equity. Counseling for lifestyle modification requires significant time, so it is difficult for general practitioners and gynecologists to spare the time needed. A study on the general practitioner’s perspective on lifestyle modification in PCOS women stated that lack of time was the primary barrier for lifestyle management from the general practitioner’s perspective [14]. A study conducted among endocrinologists and obstetricians stated that lack of access to allied health services and limited capacity for in-depth lifestyle discussions were barriers to implementing lifestyle modification [15].

The prevalence of depression in PCOS ranged from 16 to 55.6% [16]. In our study, depressive mood is expressed as one of the barriers to adhere with lifestyle modification. Lifestyle modification, including cognitive behavioral therapy, significantly improves the depressive mood and self-esteem. Self-esteem, in turn, is positively correlated with weight loss [17]. Negative body image facilitated weight reduction in our study, which is contrary to a study that stated negative body image as a barrier to engaging in physical activity [18]. Lack of motivation was found to be an additional barrier in our study, which is consistent with other studies [19,20]. Patients' motivation is deeply rooted and difficult to modify. Health professionals who engage in lifestyle management believe that patients must be responsible for lifestyle changes. But attempts taken for such changes many a time halted due to the patient’s unwillingness to change [21]. Here comes the role of patient-specific motivators, as motivational interviewing is found to be effective in smoking cessation and alcohol dependence. So, there is flourishing research regarding the role of motivational interviewing in PCOS women [22].

This study highlights (1) the need to emphasize referral to allied healthcare professionals, such as a dietitian, in facilitating adherence to lifestyle modification, which is the first line of management in PCOS, (2) in addition to the diet and exercise, cognitive behavioral therapy should also be included in the lifestyle management benefitting the depressive mood and self-esteem which is negatively correlated with the weight reduction. The 2023 guidelines recommend screening for risk factors and symptoms of depression and anxiety at the time of diagnosis [23], (3) the need for proper counseling to promote lifestyle modifications, as many women were not able to modify their lifestyle despite having good family support, which was stated as a facilitator by the other group. A key strength of this study is the simultaneous evaluation of both barriers and facilitators to lifestyle modification, including insights from women who successfully achieved weight reduction. This provides practical, patient-centered perspectives that may inform future interventions aimed at improving adherence.

However, this study has several limitations (1) being a single-center, institution-based study, the findings may not be generalizable to the wider population, particularly rural communities, as the majority of participants were from urban areas, (2) the sample size was relatively small and did not reach the initially calculated sample, which may limit statistical power, (3) the use of a self-reported questionnaire introduces the possibility of recall and reporting bias, (4) additionally, the questionnaire, although content-validated and pretested, was not a standardized instrument with formal reliability testing, (5) finally, the cross-sectional design limits the ability to establish causal relationships between identified factors and lifestyle modification outcomes, (6) lack of standardized tools for assessing depressive symptoms.

## Conclusions

This study found that adherence to lifestyle modification among women with polycystic ovary syndrome was suboptimal, with several physical and psychological factors associated with weight reduction outcomes. Depressive mood was independently associated with lower odds of achieving weight reduction, while concern about body image was associated with improved weight loss in unadjusted analyses. A notable finding was the very low utilization of dietetic services, highlighting a potential gap in multidisciplinary care. These findings suggest that nutritional counseling and psychological support may play important roles in addressing barriers to lifestyle modification in women with PCOS. Strengthening referral pathways to allied health professionals and improving patient education may help enhance adherence to lifestyle interventions. Further multicenter and interventional studies are needed to evaluate strategies that can effectively improve long-term outcomes in this population.

## Appendices

Structured questionnaire used in the study

Section A: Sociodemographic Details

Age: ______ years

Residence: ☐ Urban ☐ Rural

Education level: ☐ Uneducated ☐ Primary ☐ Secondary (6th-12th) ☐ Graduate

Occupation: ☐ Student ☐ Employed ☐ Unemployed

Socioeconomic status (Modified Kuppuswamy Scale): ☐ Lower ☐ Upper lower ☐ Lower middle ☐ Upper middle ☐ Upper

Weight: ______ kg

Height: ______ cm

Body mass index (BMI): ______ kg/m²

Section B: Disease Profile and Awareness

Symptoms (tick all that apply): ☐ Irregular menstrual cycles ☐ Infertility ☐ Obesity ☐ Acne / Alopecia / Hirsutism ☐ Others: __________

Lifestyle modification is important in the management of PCOS: ☐ Yes ☐ No ☐ Don’t know

PCOS can cause infertility: ☐ Yes ☐ No ☐ Don’t know

PCOS can lead to weight gain: ☐ Yes ☐ No ☐ Don’t know

PCOS can cause menstrual irregularity: ☐ Yes ☐ No ☐ Don’t know

PCOS can lead to diabetes mellitus: ☐ Yes ☐ No ☐ Don’t know

PCOS can cause acne or hirsutism: ☐ Yes ☐ No ☐ Don’t know

Section C: Barriers to Lifestyle Modification

Easy accessibility of junk food ☐ Yes ☐ No

Lack of time ☐ Yes ☐ No

Not adequately counseled by the doctor regarding lifestyle modification ☐ Yes ☐ No

Financial constraints to maintain a balanced diet ☐ Yes ☐ No

Lack of motivation ☐ Yes ☐ No

Feeling fatigued or sleepy ☐ Yes ☐ No

Lack of space at home for exercise ☐ Yes ☐ No

Feeling embarrassed to exercise in public (e.g., gym) ☐ Yes ☐ No

Depressive mood ☐ Yes ☐ No

Section D: Facilitators to Lifestyle Modification

Concern about body image ☐ Yes ☐ No

Support from family members ☐ Yes ☐ No

Doctor advised that 5% weight reduction improves menstrual cycles ☐ Yes ☐ No

Doctor advised that lifestyle modification improves fertility ☐ Yes ☐ No

Availability of balanced diet meals ☐ Yes ☐ No

Consultation with a dietitian ☐ Yes ☐ No

Peer support (friend undergoing lifestyle modification) ☐ Yes ☐ No
